# Treatment of Central Nervous System Tumors on Combination MR-Linear Accelerators: Review of Current Practice and Future Directions

**DOI:** 10.3390/cancers15215200

**Published:** 2023-10-29

**Authors:** John Michael Bryant, Ajay Doniparthi, Joseph Weygand, Ruben Cruz-Chamorro, Ibrahim M. Oraiqat, Jacqueline Andreozzi, Jasmine Graham, Gage Redler, Kujtim Latifi, Vladimir Feygelman, Stephen A. Rosenberg, Hsiang-Hsuan Michael Yu, Daniel E. Oliver

**Affiliations:** 1 Department of Radiation Oncology, H. Lee Moffitt Cancer Center and Research Institute, Tampa, FL 33612, USAibrahim.oraiqat@moffitt.org (I.M.O.); jacqueline.andreozzi@moffitt.org (J.A.); gage.redler@moffitt.org (G.R.); kujtim.latifi@moffitt.org (K.L.); michael.yu@moffitt.org (H.-H.M.Y.); 2 Morsani College of Medicine, University of South Florida, Tampa, FL 33602, USA; adoniparthi@usf.edu

**Keywords:** radiation therapy, RT, ultra-hypofractionated radiation therapy, ablative radiation therapy, adaptive radiation therapy, image-guided radiotherapy, magnetic resonance imaging, MRI, MR-guided radiation therapy, MRgRT, stereotactic body radiotherapy, SRS, stereotactic radiosurgery, SRT, stereotactic radiation therapy, plan optimization, tumor motion management, multiparametric MRI, mpMRI

## Abstract

**Simple Summary:**

Magnetic resonance imaging (MRI) has several advantages over computerized tomography (CT) in the treatment planning of central nervous system (CNS) malignancies. The adoption of hybrid MRI and linear accelerators (MRLs) has allowed for more effective tumor control and reduced unnecessary neurotoxicity through precise daily adaptations. In this review, we provide a summary of the evidence for MRLs in the management of various CNS tumors. Additionally, we discuss the potential of multiparametric MRI and genomically guided radiotherapy to enhance patient outcomes.

**Abstract:**

Magnetic resonance imaging (MRI) provides excellent visualization of central nervous system (CNS) tumors due to its superior soft tissue contrast. Magnetic resonance-guided radiotherapy (MRgRT) has historically been limited to use in the initial treatment planning stage due to cost and feasibility. MRI-guided linear accelerators (MRLs) allow clinicians to visualize tumors and organs at risk (OARs) directly before and during treatment, a process known as online MRgRT. This novel system permits adaptive treatment planning based on anatomical changes to ensure accurate dose delivery to the tumor while minimizing unnecessary toxicity to healthy tissue. These advancements are critical to treatment adaptation in the brain and spinal cord, where both preliminary MRI and daily CT guidance have typically had limited benefit. In this narrative review, we investigate the application of online MRgRT in the treatment of various CNS malignancies and any relevant ongoing clinical trials. Imaging of glioblastoma patients has shown significant changes in the gross tumor volume over a standard course of chemoradiotherapy. The use of adaptive online MRgRT in these patients demonstrated reduced target volumes with cavity shrinkage and a resulting reduction in radiation dose to uninvolved tissue. Dosimetric feasibility studies have shown MRL-guided stereotactic radiotherapy (SRT) for intracranial and spine tumors to have potential dosimetric advantages and reduced morbidity compared with conventional linear accelerators. Similarly, dosimetric feasibility studies have shown promise in hippocampal avoidance whole brain radiotherapy (HA-WBRT). Next, we explore the potential of MRL-based multiparametric MRI (mpMRI) and genomically informed radiotherapy to treat CNS disease with cutting-edge precision. Lastly, we explore the challenges of treating CNS malignancies and special limitations MRL systems face.

## 1. Introduction

Central nervous system (CNS) tumors pose unique and significant challenges for oncologists. As the understanding of CNS tumors continues to improve, innovative approaches and advancements in radiotherapy techniques are needed to take advantage of these insights to improve patient outcomes. The integration of magnetic resonance imaging (MRI) with linear accelerators (MRLs) has allowed for adaptive magnetic resonance-guided radiotherapy (MRgRT), which has emerged as a promising modality for increased personalization in radiation therapy for treating CNS tumors.

MRI plays a central role in both the diagnosis and evaluation of CNS tumors due to its excellent soft tissue imaging and ability to visualize the boundaries between the tumor and normal tissues [1,2]. High-resolution anatomical and parametric imaging techniques have significantly improved the accuracy of target delineation [3,4]. Thus, the integration of MRI into treatment planning has become more prevalent, leading to better disease control and fewer toxicities [5,6].

**Figure 1:**
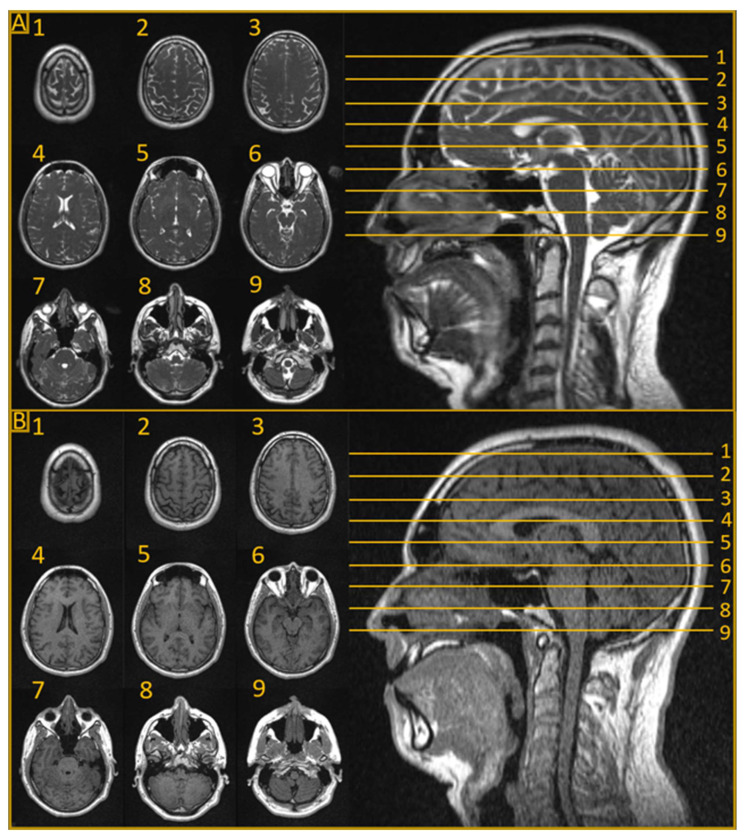
Magnetic resonance imaging (MRI) sequences on a 0.35 T combination MRI and linear accelerator (MRL) without contrast. (**A**) A true fast imaging with steady-state free precession (TRUFI) sequence of a healthy brain. The in-plane resolution and acquisition time for the TRUFI sequence were 1.5 mm and 1 min and 40 s, respectively. The TRUFI sequence provides contrast that is a combination of T_1_ and T_2_ weighting and is the sequence used for planning on the 0.35 T MRL. (**B**) T_2_ fluid-attenuated inversion recovery (FLAIR) sequence of a healthy brain. The in-plane resolution and acquisition time for the FLAIR sequence were 0.75 mm and 12 min and 6 s, respectively. Both sets of images (FLAIR and TRUFI) have a slice thickness of 1.5 mm and were acquired using a six-channel phased-array receiver head coil.

The advent of MRLs marked a revolutionary step in the field of MRgRT for CNS tumors [6,7]. In addition to their soft tissue imaging advantages, MRLs also enable adaptation of treatment plans based on daily intrafraction anatomical changes. These advantages work together to effectively expand the therapeutic window for CNS RT by improving dose delivery to the tumor and minimizing the exposure of surrounding healthy tissue [8,9]. The two commercially available MRLs are the ViewRay MRIdian (ViewRay Technologies Inc., Oakwood Village, Ohio) and Elekta Unity (Elekta AB, Stockholm, Sweden) systems [10]. These systems make up the majority of clinical MRL implementations and have been used with great efficacy to treat tumors across the body [10,11]. The MRIdian system integrates a 0.35 T field strength split-bore magnet MRI with a 6 MV flattening filter-free (FFF) linear accelerator [12], while the Elekta Unity combines a 1.5 T MRI with a 7 MV FFF linear accelerator [13]. As an example of the imaging capabilities of the low-field MRIdian unit, Figure 1 demonstrates a T_2_ fluid-attenuated inversion recovery (FLAIR) sequence and a true fast imaging with steady-state free precession (TRUFI) sequence [14] of a healthy brain. The Unity system’s conventional strength MRI generates images of similar quality to a 1.5 T diagnostic MRI unit, and the system has access to all clinically available sequences on Philips MR systems. Although both systems have their unique strengths and limitations, they share the same advantages over conventional computerized tomography (CT)-based radiation therapy in the treatment of CNS tumors. These advantages are actively being explored in several innovative trials. Table 1 lists currently active trials exploring CNS radiation therapy on an MRL that are registered on ClinicalTrials.gov (8 August 2023).

In the subsequent sections, we discuss the unique challenges and limitations associated with treating adult patients with CNS tumors using traditional radiotherapy methods and how MRgRT offers a potential solution to address these issues. Specifically, we explore the role of MRgRT in the treatment of glioblastoma (GBM) and the unique ability of MRLs to detect subtle soft tissue changes and plan adaptation to improve tumor targeting and reduce dose to surrounding healthy tissue [15]. Furthermore, we will examine the dosimetric feasibility and potential advantages of delivering stereotactic radiotherapy (SRT) on an MRL for intracranial [16,17,18,19,20] and spine [21,22,23] lesions. We review recent studies and highlight the ongoing clinical trials that aim to further evaluate the clinical benefits and challenges of these approaches. We also discuss the potential role of MRL-based hippocampal avoidance whole brain radiation therapy (HA-WBRT), a promising technique that strives to balance effective tumor control and minimize neurotoxicity for patients undergoing WBRT [24,25,26]. By providing enhanced target delineation and facilitating daily adaptive treatment to account for therapeutic tumor response throughout therapy, MRLs demonstrate potential to optimize HA-WBRT [15,27]. Additionally, we discuss the future direction of biologically adaptive radiation therapy for CNS tumors and provide a general introduction to how multiparametric MRI (mpMRI) and genomics may pave the path towards this paradigm shifting approach [28,29,30,31]. Finally, we will review the limitations and challenges that MRLs have in general, as well as the specific challenges in the treatment of CNS tumors [32,33].

## 2. Clinical Applications

### 2.1. Glioblastoma

In accordance with the guidelines established by the Radiation Therapy Oncology Group (RTOG), the postoperative GBM target includes both the surgical cavity and the adjacent edema [34]. This approach is supported by autopsy data [35] and stereotactic biopsies [36], which reveal tumor cells infiltrating up to and beyond the edges of FLAIR hyperintensity. As a result, target volumes are generated using preoperative and postoperative MRI to delineate the tumor resection cavity, residual enhancement, and surrounding edema [37]. However, these features continue to evolve postoperatively and during radiation treatment, leading to discrepancies between the actual and pre-RT anatomy [37,38]. 

Although conducting MRI scans throughout RT to account for these changes would be ideal, it is not feasible due to limited scanner availability and cost constraints. Unfortunately, CT-guided radiotherapy (CTgRT) does not adequately account for parenchymal changes because of its inadequate soft tissue contrast. MRLs may offer a solution, as they allow for superior soft tissue contrast to detect subtle geometric changes and for online plan adaptation to account for tumor and post-operative bed changes during therapy. The need for geometric plan adaptation is highlighted in a prospective study involving 61 GBM patients who underwent chemoradiotherapy (CRT) and received diagnostic brain MRIs at planning, fraction 10, fraction 20, and one-month post-CRT. Significant anatomical changes were observed throughout therapy [39]. The study demonstrated that targets experienced both changes in volume and migration throughout treatment [39]. Furthermore, a case series of three GBM patients who underwent standard CRT on an MRL demonstrated significant changes in edema and cavity volume throughout treatment [40]. 

A recent study investigated the potential benefits of adaptive radiotherapy for GBM patients using an MRL system [15]. The study evaluated the dosimetric differences between non-adapted versus weekly plan-adapted RT of GBM patients treated with CRT of 60 Gy on a 0.35 T MRL. Weekly plan adaptation demonstrated mean improvements in uninvolved hippocampi and normal brain dose of 8.4 versus 12.5 Gy and 18.7 (*p* = 0.036) versus 20.6 Gy (*p* = 0.005), respectively [15]. Multiple additional studies summarized in Table 2 demonstrate how MRLs can be leveraged to account for GBM target evolution throughout therapy to improve target coverage and spare normal tissue.

In summary, treatment of GBMs with MRLs is associated with improvements in dosimetry and treatment planning due to its ability to detect subtle soft tissue changes and facilitate plan adaptation. Additionally, MRLs can account for changes in the tumor and tumor bed throughout treatment to ensure better target coverage while minimizing unnecessary dose to normal brain parenchyma. Future studies are needed to determine if this translates into better disease control durability and if better normal tissue sparing results in quality-of-life improvements for these patients.

### 2.2. Stereotactic Radiation Therapy for Intracranial Tumors

SRT is an important modality in the management of brain metastases and other intracranial tumors [45]. An MRL offers several advantages over conventional linear accelerators, including enhanced target delineation, real-time tumor tracking, and adaptive treatment planning [10]. The superior soft-tissue contrast provided by MRI allows for improved identification of tumor boundaries, while real-time imaging enables accurate monitoring of tumor motion during treatment, potentially reducing unnecessary radiation exposure to healthy brain tissue [46].

Several studies have explored the dosimetric feasibility of MRgRT for intracranial SRT [16,17,18,19,20]. One early study investigated the dosimetric feasibility of MRL for brain metastases and the impact of the magnetic field, concluding that MRL-generated stereotactic radiation plans meeting clinical requirements were feasible, and that the dosimetric impact of the magnetic field, including the electron return effect (ERE) at tissue–air boundaries, was minor and did not negatively affect target conformity or dose gradient [16]. Another study evaluated the systematic localization accuracy, treatment planning capability, and delivery accuracy of an MRL platform for stereotactic radiosurgery (SRS), finding that excellent plan quality and delivery accuracy was achievable for concurrent treatment of multiple brain metastases with a single isocenter [17]. A comparative study assessed the dosimetric feasibility of brain SRT, comparing a 0.35 T MRL and a conventional linear accelerator. It was revealed that the MRL could generate clinically acceptable SRS plans for spherical intracranial lesions with a diameter ≤ 2.25 cm [18]. However, larger lesions (>2.25 cm) did not meet normal tissue dosimetric constraints [18]. Additionally, caution and extra thought should be given when treating patients who have undergone systemic therapies that may exhibit pseudo-progression/treatment effect phenomena [47] between simulation and treatment, as these can falsely increase the volumetric appearance and lead to excess normal tissue doses.

Another study examined the dosimetric feasibility of direct post-operative MRL-based SRT for resection cavities of brain metastases, concluding that direct post-operative MRL-based SRT is dosimetrically acceptable, with benefits including increased patient comfort and logistics [19]. Streamlining workflow in this way may prove beneficial in the setting of postoperative SRT, given that delay in the delivery of radiotherapy following resection of brain metastases has been associated with a decrement in local control [48]. The study suggests that the clinical benefit of this workflow should be investigated given its dosimetric plausibility. Lastly, a study investigated changes in the clinical target volume (CTV) and associated clinical implications of an MRL during hypofractionated SRT to resected brain metastases, finding statistically significant reductions in cavity CTV during SRT, which supports the use of MRgRT and treatment adaptation to mitigate toxicity [20]. Another potential application of MRgRT is in preoperative SRT, a modality which has recently gained traction, particularly for larger tumors [49]. Particularly in patients who may have symptomatic brain metastases, carefully planned use of MRgRT may expedite treatment and facilitate a shorter interval from diagnosis through resection.

Collectively, the above studies indicate that MRgRT using an MRL has the potential to offer dosimetric and logistical advantages over conventional linear accelerators for intracranial SRT treatment. However, further clinical investigations are necessary to evaluate the clinical benefit of this technology.

### 2.3. Stereotactic Radiation Therapy for Spine Tumors

Spine SRT plays a critical role in managing metastatic disease by alleviating pain, preventing pathological fractures, and reducing neurological morbidity. Stereotactic body radiotherapy (SBRT) has been shown to provide improved efficacy compared with conventional radiotherapy methods [50]. For spine SBRT, fusion with either a planning MRI or CT myelogram is necessary to accurately delineate the spinal cord and establish a 1–2 mm planning organ-at-risk volume (PRV), potentially reducing disease coverage [51]. CTgRT relies on bony structures for daily setup; however, it cannot visualize the spinal cord, leading to the requirement of a cord PRV for daily motion management.

MRLs offer several advantages over CTgRT, including MR imaging in treatment position to allow for easier fusion with the planning CT and superior spinal cord delineation during setup compared with cone-beam CT (CBCT) [21,23]. Dosimetric feasibility studies indicate that daily MRgRT can lower the dose to the spinal cord [22]. MRLs enable daily direct registration to the spinal cord, eliminating the need for cord PRVs while also allowing for improved tumor coverage with reduced margin size. In addition, low-field MRLs suffer from less image distortion from implanted metallic devices [52]. MRI protocols outside of the radiation oncology department can often create challenges to performing an accurate fusion due to differences in slice thicknesses and patient positions [53]; simulation and treatment planning using MRLs may address these issues [23]. These advantages increase the therapeutic ratio and may allow for dose escalation.

Several clinical trials are currently investigating the use of MRL in spine SRS/SBRT. These studies will provide valuable insights into the feasibility and effectiveness of this technique for spine treatment. For example, a phase I/II trial is examining the use of Stereotactic MR-guided Adaptive Radiotherapy (SMART) for treating various disease sites, including the spine (NCT04115254). Additionally, the Pilot Study of Same-session MR-only Simulation and Treatment with SMART for Oligometastases of the Spine (NCT03878485) focuses specifically on spine treatment.

As more clinical data emerge from these studies, the potential benefits of MRL in spine SRT will become clearer. The integration of MRI with linear accelerators for spine treatment may lead to improved tumor targeting, reduced normal tissue exposure, and better patient outcomes. Ultimately, these advancements may help optimize radiotherapy for patients with metastatic spine disease and contribute to the ongoing evolution of spine SRT techniques.

### 2.4. Hippocampal Avoidance Whole Brain Radiotherapy

HA-WBRT is a promising technique that aims to achieve the local control benefits of whole brain RT for both macro- and micro-metastatic lesions, while reducing neurotoxicity by specifically avoiding the hippocampus [54]. HA-WBRT requires fusion of a planning MRI with CT simulation imaging to create a hippocampal avoidance structure, while ensuring a homogenous treatment plan for the remaining brain parenchyma [55]. HA-WBRT using MRgRT could facilitate adaptive treatment based on the intra-therapeutic tumor response, potentially improving the local control probability with sequential stereotactic boosts to nonresponding lesions. A dosimetric feasibility study compared twelve HA-WBRT volumetric modulated arc therapy (VMAT) plans versus intensity modulated radiation therapy (IMRT) plans created using the 0.35 T MRL treatment planning system (TPS) [27]. In all cases, the researchers were able to generate plans that met RTOG 0933 treatment plan standards. As anticipated, the VMAT plans exhibited superior homogeneity and delivery times compared with the IMRT plans.

Particularly for patients with numerous brain metastases of radioresistant histology, HA-WBRT with a simultaneous integrated boost to gross disease may be an effective strategy [14,56], one that may be further facilitated using MRgRT. Additional investigation through clinical trials is necessary to determine the clinical feasibility and safety of HA-WBRT. This approach could optimize the balance between effective tumor control and minimizing neurotoxicity for patients undergoing whole brain radiotherapy.

## 3. Future Directions

One of the fundamental goals of radiotherapy is maximizing the dose to target tissue while minimizing the dose to surrounding OARs. In a significant first step towards this aim, MRLs have facilitated treatment plan adaptation to observable anatomic changes throughout therapy. MRLs, however, may be able to enable biological plan adaptation by leveraging MRI’s capability to track biological and physiological changes through advanced mpMRI techniques. These techniques may allow radiation oncologists further insight into a tumor’s biology as it responds to RT over the course of treatment.

The treatment paradigm of radiotherapy has traditionally been based on empirical large cohort data rather than individual biology, resulting in a one-size-fits-all approach. However, recent advancements in genomics and radiomics have begun to pave the way towards a more personalized approach based upon individual tumor biology. The synergy between genomically informed radiotherapy, treatment of high-risk sub-volumes based on extraction of radiographic data, and daily mpMRI-guided plan adaptation has the potential to usher in a new treatment paradigm in radiation oncology. Pre-treatment genomic and radiomic analyses of the tumor may improve patient selection for MRL-based dose escalation [30,57,58]. Daily mpMRI delta radiomic analysis can then be used to detect subtle biomarkers of treatment response in tumors, which hint at radiation-induced genomic plasticity, thereby allowing for even greater personalized adaptive treatment strategies [59,60,61,62].

The currently active phase II Habitat Escalated Adaptive Therapy (HEAT), With Neoadjuvant Radiation for Soft Tissue Sarcoma (NCT05301283) is an example of a cutting-edge study utilizing genomic and mpMRI radiomic biomarkers to guide the initial treatment and adaptive treatment approach for high-grade soft tissue sarcomas. Utilizing a similar approach for CNS tumors appears to be technically feasible currently. For example, genomic-adjusted radiation therapy (GARD) could be similarly utilized to identify GBM patients who could benefit from higher doses [63] with MRI perfusion [64] and FLAIR [65,66] sequences, which could identify tumor subpopulations to target with simultaneous integrated boosts.

MRL is poised to take a central role at the forefront of this paradigm shift to allow for plan adaptation based not only upon geometric shifts but also on a tumor’s evolving treatment response throughout therapy. An ultra-personalized treatment approach like this allows for total dose, dose distribution (i.e., dose painting), and fractionation changes throughout the course of therapy to improve clinical outcomes for patients. 

### Radiomic- and Genomic-Guided Adaptive Radiation Therapy for CNS Tumors

Historically, daily MRgRT plan adaptation has been utilized to manage interfractional geometric changes. However, MRI is also capable of assessing biological and physiological information using advanced mpMRI techniques [28,67,68,69]. These techniques have the potential to be particularly beneficial for CNS tumors treated on an MRL.

One such technique is diffusion-weighted imaging (DWI), which enables the detection of water mobility changes [70]. These alterations are associated with tumor growth [71] or necrosis [72]. By mapping a parameter known as the apparent diffusion coefficient (ADC), clinicians can monitor the response to radiation therapy [73]. ADC mapping is especially appealing in adaptive radiotherapy, as changes in ADC can be observed before morphological alterations in the tumor [74]. This feature could guide dose escalation strategies or biologically driven radiation plan adaptation [58,75]. DWI has been applied on a 1.5 T MRI-guided linear accelerator [76,77,78] and on a 0.35 T tri-cobalt system [79,80]. When the 0.35 T MRgRT system transitioned from a tri-cobalt system to an MRL, technical challenges emerged [81]. However, recent applications of DWI on a 0.35 T MRL show promise [10].

Dynamic contrast-enhanced (DCE) MRI is another functional imaging technique that investigates perfusion by dynamically evaluating changes in the T_1_ relaxation time following a bolus injection of gadolinium [82,83]. This process allows for the examination of gadolinium transport across the capillary endothelium [68]. DCE has demonstrated prognostic value in identifying patient subpopulations with hypoxia-related resistance to chemoradiation in cervical cancer [84]. As a result, DCE has the potential to provide information that may aid in personalizing radiation dose delivery for CNS tumors [77]. DCE has been implemented on the 1.5 T MRL, although its quantification was shown to be less reproducible than that of T_1_, T_2_, and ADC [76]. While DCE has not yet been implemented on a 0.35 T MRL, initial experiences have demonstrated the feasibility of gadolinium injection on the tri-cobalt version of this system [85].

Additional MR-based techniques, such as magnetic resonance spectroscopic imaging (MRSI) [86], chemical exchange saturation transfer (CEST) [87,88], and hyperpolarized dynamic magnetic resonance spectroscopy [89], can interrogate metabolic processes in tumors [90]. These techniques could potentially offer valuable information for the adaptive radiotherapy of CNS tumors on an MRL. MRSI has been applied to create high-resolution metabolite maps in gliomas [91] and to map lactate in GBM [92]. Furthermore, MRSI has been employed to map intra- and extracellular pH in tumors using phosphorus [93]. However, integrating MRSI into online MRgRT still faces technical limitations, such as relatively long scan times [94] and low sensitivity on conventional magnetic field strength systems [95]. Hyperpolarizing the nucleus can counteract the low sensitivity, resulting in a significant gain in sensitivity for a short period of time [96]. The primary application of hyperpolarization has been to observe the dynamic conversion of pyruvate into lactate in tumors [97]. CEST, on the other hand, enables the indirect detection of low-concentration solutes via their effect on the water MR signal [98]. CEST has been shown to predict chemoradiotherapeutic response in tumors [29,99]. Moreover, it has been shown to be capable of noninvasively determining IDH mutation and MGMT methylation status in vivo [100]. While these techniques show promise, further development and optimization are necessary to fully harness their potential in guiding adaptive radiotherapy for CNS tumors on an MRL.

Although none of the CNS-specific trials within Table 1 have reported results yet, the multi-disease site “MR-BIO: A Study to Evaluate Changes in MR Imaging and Biological Parameters (MR-BIO)” (PMID: NCT04903236) have published results within their head and neck patient cohort which demonstrated that a 1.5 T MRL can reliably be used to detect tumor hypoxia via oxygen-enhanced MRI (OE-MRI) [101]. OE-MRI is another example of a powerful MR technique that detects oxygenation in both normal tissues and tumors after inhalation of 100% oxygen or carbogen, which allows for the quantification and mapping of hypoxia over the course of radiotherapy [102,103,104,105].

Genomics provides another powerful avenue towards personalized radiation treatment, which when combined with the advances in image guidance listed above, may provide an even more sophisticated approach for challenging malignancies such as GBM. Several such signatures have been introduced as a potential means for genomically guided RT [57,63,106,107,108,109,110]. Genomics offers a biological framework for guiding RT, giving better context to the radiomic changes observed during treatment, and fostering research into predictive biomarkers. This combination may allow for novel advanced and better-informed approaches to dose escalation in high-grade glioma, a strategy which has been largely unsuccessful in the past [111,112,113,114]. 

In summary, MRL can be leveraged by incorporating genomically guided RT and mpMRI radiomics to enable a biologically adaptive RT paradigm. Various mpMRI techniques, including DWI, DCE, MRSI, and CEST, have the potential to offer valuable insights into tumor biology and physiology, ultimately leading to more personalized and effective treatment strategies. Using these technologies to identify intratumoral heterogeneity and tumoral sub-volumes at risk may allow for focal dose escalation or avoidance, respectively. As research and development continues in this area, we expect significant advancements in the application of these techniques, potentially revolutionizing the management of CNS tumors.

## 4. Barriers and Limitations in CNS Radiation Therapy on MRL

### 4.1. General Limitations

MRgRT offers significant advancements for image-guided radiation therapy (IGRT) and personalized oncology in CNS tumor treatment, but there are certain limitations to be considered. These include substantial financial and time investments for training and operation, development of MR-safety protocols, and unique physical challenges related to the concurrent use of MR and external beam radiotherapy [115,116,117]. Additionally, the daily online adaptive radiotherapy process can be time-consuming, and patient selection is crucial, as some patients may have difficulty tolerating the treatment due to claustrophobia, large body habitus, or MR-incompatible implanted devices [118].

### 4.2. Unique Challenges in Treating CNS Tumors on an MRL

Treating CNS tumors on an MRL presents several unique challenges. The complex anatomy of the CNS, with its highly radiosensitive normal tissues, requires precise targeting and dose delivery. The proximity of critical structures, such as the optic nerves, brainstem, and spinal cord, demands meticulous treatment planning and delivery. Furthermore, CNS tumors often exhibit infiltrative growth patterns, making it almost impossible to accurately delineate tumor boundaries.

MRL systems suffer the same limitations as diagnostic MRIs. As such, they’re sensitive to the magnetic susceptibility artifacts that arise from air–tissue [119] and bone–tissue [120] interfaces in the skull base, sinuses, and mastoid air cells, which can affect image quality and accuracy for CNS tumor localization. Moreover, the blood–brain barrier (BBB) influences the performance of MRI techniques, such as DCE MRI. The BBB’s integrity may affect the permeability of gadolinium-based contrast agents and, consequently, the accuracy of DCE-derived parameters [121]. These challenges require further research and the development of advanced MRI techniques to address them.

Geometric distortion represents one of the most difficult challenges to account for with any MRgRT. Geometric distortion occurs due to imperfections in the magnetic field, gradient nonlinearities, and magnetic susceptibility differences at tissue interfaces [122]. This can lead to inaccuracies in the target delineation and treatment planning of CNS tumors. Additionally, geometric distortion can make it difficult to accurately account for small radiosensitive structures, such as the optic nerves, cochlea, pituitary gland, and hippocampus. Both 1.5 T and low-field MR systems experience varying degrees of geometric distortion. Typically, 1.5 T MR systems exhibit larger distortions than low-field systems due to larger field inhomogeneities and increased susceptibility effects at higher field strengths [120]. However, even low-field MR systems may present geometric distortion challenges, particularly at air–tissue interfaces and near metal implants. Addressing the issue of geometric distortion in MRgRT for CNS tumors requires further development of advanced MRI techniques and correction algorithms.

## 5. Conclusion

Treatment of CNS tumors on MRLs represents a promising direction for the advancement of personalized cancer care. MRLs provide excellent soft tissue visualization, real-time monitoring of targets and normal tissues with gating capabilities, and the ability for daily plan adaptation. These unique advantages hold the potential to improve radiation delivery to patients with intracranial or spine tumors. Clinical trials are currently underway and seek to clarify the role of MRLs in the treatment of CNS malignancies. Future clinical studies are needed to explore the integration of mpMRIs and genomics to develop a biologically adapted radiation therapy paradigm for CNS tumors.

## Figures and Tables

**Table 1:** Ongoing MRL CNS clinical trials registered on ClinicTrials.gov (8 August 2023).

Study Title	Sponsor	Site	Condition/Disease	Estimated Enrollment	Identifier
MR-Linac Guided Adaptive FSRT for Brain Metastases From Non-small Cell Lung Cancer	Sun Yat-sen University	CNS	Brain Metastases from Non-small Cell Lung Cancer	55	NCT04946019
UNIty-Based MR-Linac Guided AdapTive RadiothErapy for High GraDe Glioma: A Phase 2 Trial (UNITED)	Sunnybrook Health Sciences Centre	CNS	Glioma, High grade	97	NCT04726397
UNIty-Based MR-Linac Guided Adaptive RadioThErapy for High GraDe Glioma-3 (UNITED-3)	Sunnybrook Health Sciences Centre	CNS	Glioma, High grade	40	NCT05720078
Pilot Study of Same-session MR-only Simulation and Treatment With Stereotactic MRI-guided Adaptive Radiotherapy (SMART) for Oligometastases of the Spine	Washington University School of Medicine	CNS	Oligometastases of the Spine	10	NCT03878485
Response Assessment During MR-guided Radiation Therapy for Glioblastoma (MARGA)	University of Zurich	CNS	Glioblastoma	20	NCT05565326
Phase II Cohort of Spinal Stereotactic Radiotherapy in Patients Using a MR LINAC	M.D. Anderson Cancer Center	CNS	Spinal Disease	40	NCT05709782
UNITy-BasED MR-Linac Adaptive Simultaneous Integrated Hypofractionationed Boost Trial for High Grade Glioma in the Elderly (UNITED2)	Sunnybrook Health Sciences Centre	CNS	Glioblastoma	55	NCT05565521
Prospective Evaluation of Radiotherapy Using Magnetic Resonance Image Guided Treatment (PERMIT)	Institute of Cancer Research, United Kingdom	All/Multiple Sites	N/A	200	NCT03727698
PRIMER: Development of Daily Online Magnetic Resonance Imaging for Magnetic Resonance Image Guided Radiotherapy	Institute of Cancer Research, United Kingdom	All/Multiple Sites	N/A	173	NCT02973828
MR-BIO: A Study to Evaluate Changes in MR Imaging and Biological Parameters (MR-BIO)	University of Manchester	All/Multiple Sites	N/A	250	NCT04903236
Imaging Acquisition and Analysis Methods for Optimization of MRI Radiation Oncology Simulation and Response Assessment	Memorial Sloan Kettering Cancer Center	All/Multiple Sites	N/A	447	NCT02422550
Northern Alberta Linac-MR Image-Guided Radiotherapy (Northern LIGHTs-2)	AHS Cancer Control Alberta	All/Multiple Sites	N/A	112	NCT05413473
Magnetic Resonance-Guided Adaptive Radiotherapy (MRgART) Using an Integrated Magnetic Resonance Linear Accelerator (MRL)	University Health Network, Toronto	All/Multiple Sites	N/A	500	NCT04135794
Stereotactic Magnetic Resonance Guided Radiation Therapy	Dana-Farber Cancer Institute	All/Multiple sites	N/A	1000	NCT04115254
The MR-Linac Technical Feasibility Protocol (UMBRELLA-II)	The Netherlands Cancer Institute	All/Multiple sites	N/A	140	NCT04351204
Solid Tumor Imaging MR-Linac (STIM Study)	Medical College of Wisconsin	All/Multiple sites	N/A	295	NCT03500081
Feasibility of Online MR-guided Radiotherapy on a 1.5T MR-Linac	University Hospital Tübingen	All/Multiple sites	N/A	472	NCT04172753
The MOMENTUM Study: The Multiple Outcome Evaluation of Radiation Therapy Using the MR-Linac Study (MOMENTUM)	University Medical Center Utrecht	All/Multiple sites	N/A	6000	NCT04075305

Abbreviations. CNS: central nervous system; N/A: not applicable.

**Table 2:** MRL GBM Studies.

Study	Type of Study	*n*	MRL Field Strength	Endpoints	Results	Conclusions
Guevara et al. 2023. [15]	Retrospective	10	0.35 T	Dose to hippocampi and normal brain parenchyma	Doses to hippocampi for static vs. weekly adaptive plans were max 21 ± 13.7 Gy vs. 15.2 ± 8.2 Gy (*p* = 0.003), mean 12.5 ± 6.7 Gy vs. 8.4 ± 4.0 Gy (*p* = 0.036), respectively. The mean brain dose was 20.6 ± 6.0 Gy for static planning vs. 18.7 ± 6.8 Gy for weekly adaptive planning (*p* = 0.005).	Weekly adaptive MRgRT replanning of shrinking resection cavity may decrease risk of RT-induced neurotoxicity by potentially sparing normal brain and hippocampi from high-dose radiation.
Jones et al. 2020.* [41]	Retrospective	14	0.35 T	MRI volumetric changes during RT	4 of 14 patients had ≥25% increase in T_2_ hyperintense volume that correlated with both T_2_/FLAIR and contrast-enhanced volume expansion on post-RT diagnostic MRIs. Patients with growth during therapy exhibited excellent survival	MRgRT may help to identify early pseudoprogression in GBM based on T_2_-weighted volume increases.
Tseng et al. 2022. [42]	Retrospective	10	1.5 T	MRL treatment characteristic and feasibility	3 patients had re-planning due to progression of disease identified on daily MRL imaging. The median ADC within the FLAIR hyper intense region and the volumes of T_2_ FLAIR were correlated (R = 0.68, *p* < 0.001).	MRgRT is feasible for high-grade gliomas. The adapt-to-position workflow and treatment times were clinically acceptable, and daily online MRL imaging triggered adaptive re-planning for selected patients. Acquisition of mpMRIs was feasible on the MRL during routine treatment workflow.
Wang et al. 2022. [43]	Retrospective	37	1.5 T	Dosimetric impact of MRL magnetic fields	MRL plans had 1.52 Gy higher mean dose to air cavities (*p* < 0.0001) and 1.10 Gy higher mean dose to skin (*p* < 0.0001).	MRL magnetic fields have minimal dosimetric impact for target volumes and standard OARs; higher doses to tissues at skin surface and surrounding air cavities in comparison to conventional Linac.
Mehta et al. 2018. [40]	Retrospective	3	0.35 T	MRL imaging dynamics throughout RT	A general trend of daily decreases in cavity measurements was observed in all patients.	Daily MRL imaging is able to detect volume changes during RT.
Cullison et al. 2022. [44]	Retrospective	34	0.35 T	MRL imaging dynamics throughout RT	The margin required to avoid the average lesion from growing out of margins from PL was 1.30 cm (max 4.1 cm). Adapting to a shrinking RC saved 26.92 mL (9.66–58.63 mL) of normal appearing brain from full RT dose.	Clinically significant anatomic changes were seen in GBM patients during CRT. Patients with unresected lesions require large RT margins or volume expansions for growth during RT. As RCs shrink, margins can be reduced to save normal brain.
Stewart et al. 2021. [39]	Prospective	61	1.5 T	MRL imaging dynamics throughout RT	The GTV migration distances were >5 mm in 46% (54%) of patients at Fx10, 50% (58%) of patients at Fx20, and 52% (57%) of patients at 1 month after therapy. Dynamic tumor morphologic changes were observed, with 40% of patients exhibiting a decreased GTV (i.e., volume relative to PL ≤ 1) with a migration distance > 5 mm during chemoradiation therapy.	Daily MRL imaging can identify interfraction tumor dynamics, including decrease in gross tumor volume as well as volume migration.

Abbreviations. ADC: apparent diffusion coefficient; CRT: chemoradiation therapy; FLAIR: fluid-attenuated inversion recovery; Fx: fraction; GBM: glioblastoma; GTV: gross tumor volume; MRgRT: magnetic resonance-guided radiation therapy; MRL: combination magnetic resonance imager and linear accelerator; OAR: organs at risk; OS: overall survival; PFS: progression free survival; PL: planning scan; RC: resection cavity; RT: radiation therapy; T: Tesla; * Not a peer-reviewed study.
